# Supplementary material for: Unfavourable sedentary and physical activity behaviour before and after retirement: a population-based cohort study
Source: BMJ Open. 2020 Jul 28;10(7):e037659. doi: 10.1136/bmjopen-2020-037659 (PMC7389486; doi:10.1136/bmjopen-2020-037659)
Supplement: Author's manuscript [file bmjopen-2020-037659.draft_revisions.pdf]

BMJ Open is committed to open peer review. As part of this commitment we make the peer review history of every article we publish publicly available.

When an article is published we post the peer reviewers' comments and the authors' responses online. We also post the versions of the paper that were used during peer review. These are the versions that the peer review comments apply to.

The versions of the paper that follow are the versions that were submitted during the peer review process. They are not the versions of record or the final published versions. They should not be cited or distributed as the published version of this manuscript.

BMJ Open is an open access journal and the full, final, typeset and author-corrected version of record of the manuscript is available on our site with no access controls, subscription charges or pay-per-view fees (http://bmjopen.bmj.com).

If you have any questions on BMJ Open's open peer review process please email info.bmjopen@bmj.com

Unfavourable sedentary and physical activity behaviour before and after retirement. A population-based cohort study

Journal:	*BMJ Open*
Manuscript ID	bmjopen-2020-037659
Article Type:	Original research
Date Submitted by the Author:	11-Feb-2020

Complete List of Authors:	ter Hoeve, Nienke; Erasmus Univ, Rehabilitation Medicine<br>Ekblom, Maria; Swedish School of Sport and Health Sciences<br>Galanti, Maria; Karolinska Institutet<br>Forsell , Yvonne; Karolinska Institutet,<br>Nooijen, Carla; Swedish School of Sport and Health Sciences,

Keywords:	PUBLIC HEALTH, EPIDEMIOLOGY, PREVENTIVE MEDICINE

SCHOLARONE™
Manuscripts

I, the Submitting Author has the right to grant and does grant on behalf of all authors of the Work (as defined in the below author licence), an exclusive licence and/or a non-exclusive licence for contributions from authors who are: i) UK Crown employees; ii) where BMJ has agreed a CC-BY licence shall apply, and/or iii) in accordance with the terms applicable for US Federal Government officers or employees acting as part of their official duties; on a worldwide, perpetual, irrevocable, royalty-free basis to BMJ Publishing Group Ltd ("BMJ") its licensees and where the relevant Journal is co-owned by BMJ to the co-owners of the Journal, to publish the Work in this journal and any other BMJ products and to exploit all rights, as set out in our licence.

The Submitting Author accepts and understands that any supply made under these terms is made by BMJ to the Submitting Author unless you are acting as an employee on behalf of your employer or a postgraduate student of an affiliated institution which is paying any applicable article publishing charge ("APC") for Open Access articles. Where the Submitting Author wishes to make the Work available on an Open Access basis (and intends to pay the relevant APC), the terms of reuse of such Open Access shall be governed by a Creative Commons licence – details of these licences and which Creative Commons licence will apply to this Work are set out in our licence referred to above.

Other than as permitted in any relevant BMJ Author's Self Archiving Policies, I confirm this Work has not been accepted for publication elsewhere, is not being considered for publication elsewhere and does not duplicate material already published. I confirm all authors consent to publication of this Work and authorise the granting of this licence.

For peer review only

Unfavourable sedentary and physical activity behaviour before and after retirement. A population-based cohort study

Nienke ter Hoeve, Maria M. Ekblom, Rosaria Galanti, Yvonne Forsell, Carla F.J. Nooijen

Dr. Nienke ter Hoeve
Capri Cardiac Rehabilitation, Rotterdam, the Netherlands
The Department of Rehabilitation Medicine, Erasmus University Medical Center, Rotterdam, the Netherlands
n.terhoeve@erasmusmc.nl

Dr. Maria M. Ekblom
The Swedish School of Sport and Health Sciences (GIH), Stockholm, Sweden
The Department of Neuroscience, Karolinska Institutet, Stockholm, Sweden
maria.ekblom@gih.se

Prof. Rosaria Galanti
The Department of Public Health Sciences, Karolinska Institutet, Stockholm, Sweden
Centre for Epidemiology and Community Medicine, Stockholm County Council, Sweden
Rosaria.galanti@ki.se

Prof. Yvonne Forsell
The Department of Public Health Sciences, Karolinska Institutet, Stockholm, Sweden
Centre for Epidemiology and Community Medicine, Stockholm County Council, Sweden
Yvonne.Forsell@ki.se

Dr. Carla F.J. Nooijen (Corresponding author)
The Swedish School of Sport and Health Sciences (GIH), Stockholm, Sweden
The Department of Public Health Sciences, Karolinska Institutet, Stockholm, Sweden
carla.nooijen@gih.se

Corresponding author:
Carla Nooijen
Swedish School of Sport and Health Sciences (GIH)
Lidingövägen 1
Box 5626
SE-114 86 Stockholm
carla.nooijen@gih.se

Acknowledgements
Carla Nooijen is supported by a grant from The Swedish Research Council for Health, Working Life and Welfare: FORTE (2017-01385).

For peer review only - http://bmjopen.bmj.com/site/about/guidelines.xhtml

Abstract

Background: During transition to retirement there is often a re-arrangement of daily life which might provide a key opportunity for interventions to promote a non-sedentary and active lifestyle. To be able to design effective interventions, it is essential to know which sedentary and physical behaviour domains (e.g. at home or during leisure time) have potential to facilitate healthy aging during the retirement transition.

Objective: To determine whether unfavourable sedentary and physical activity behaviour before retirement predict unfavourable sedentary and physical activity behaviour after retirement.

Design: Population-based cohort

Setting and participants: Adults (n=3272) employed in 2010 but retired in 2014.

Methods: Self-reported pre-retirement job activity, sedentary leisure time, physical activity at home, walking-cycling and exercise, were assessed as predictors for unfavourable sedentary and physical activity behaviours after retirement using logistic regression. Unfavourable behaviours were defined based on the respective median of the cohort distribution. Furthermore, the odds ratio (OR) for having multiple unfavourable behaviours post-retirement was determined, based on the amount of unfavourable behaviours pre-retirement. All models were adjusted for gender and education.

Results: Unfavourable sedentary and physical activity in a certain domain before retirement was the strongest predictor of the same behaviour after retirement. Unfavourable job activity did not predict physical activity but did predict unfavourable sedentary behaviour after retirement (OR=1.66, 95% Confidence interval (CI)=1.41-1.96). Unfavourable exercise behaviour pre-retirement predicted unfavourable sedentary and physical activity post-retirement in all domains. With all behaviours being unfavourable pre-retirement, the OR of having at least 3 unfavourable behaviours post-retirement was 36.7 (95% CI=16.8-80.5).

Conclusions: Adults with a higher number of unfavourable pre-retirement physical activity and sedentary behaviours are likely to carry these unfavourable behaviours into retirement age. Pre-retirement exercise interventions may have great potential to improve physical activity and sedentary behaviours and thereby facilitate healthy aging.

For peer review only - http://bmjopen.bmj.com/site/about/guidelines.xhtml

Strengths and limitations of the study

- The study described a large longitudinal cohort investigating changes in sedentary behaviour and physical activity during retirement transition.
- Our methods are unique in studying a large variety of behaviours related to both sedentary behaviour and physical activity in different domains (e.g. at home, during leisure time) both before and after retirement.
- This study adds with valuable knowledge for public health researchers and policy makers that of all sedentary behaviour and physical activity domains, leisure time exercise seems to have the greatest potential in pre-retirement interventions that aim to facilitate healthy aging.
- Since there is no evidence for cut-off points for domain-specific physical activity and sedentary behaviour, we used the sample medians to distinguish between favourable and unfavourable behaviour.
- We did not have information on the exact time since retirement.

Funding statement

The work of Carla Nooijen was supported by The Swedish Research Council for Health, Working Life and Welfare grant number [FORTE (2017-01385)].

Competing interests: None declared.

Author's contribution

All authors contributed to the design, methodology, and to manuscript review. CFJN was responsible for data analysis. NtH and CFJN were responsible for drafting the manuscript. All authors read and approved the final manuscript.

Data sharing

Applications for access to data can be send to the Stockholm County Council https://www.folkhalsoguiden.se/halsa-stockholm/halsa-stockholm---for-forskare/

Patient and public involvement

Patients or the public were not involved in the design, or conduct, or reporting, or dissemination plans of our research

For peer review only

Introduction

An aging population and increasing life expectancy result in a growing number of adults spending a long time in retirement.[1] Sedentary behaviour and physical activity are two related and independent predictors of healthy aging.[2] More sedentary time and less physical activity are related to increased risk of diabetes, cardiovascular disease, and all-cause mortality.[3-7] Studies in older adults have additionally shown that unfavourable sedentary and physical activity patterns increase the risk of functional limitations in the performance of activities of daily life such as walking and performing house chores.[8]

Elderly spend 65-80% of their waking hours sedentary and only a minority meets physical activity recommendations.[9 10] During transition to retirement, daily life often undergoes a re-arrangement which might provide a key opportunity for interventions to stimulate a non-sedentary and active lifestyle. Lack of time is a frequently reported barrier to physical activity, which might not exist any longer after retirement.[11]

There is a lack of well-conducted studies investigating changes in sedentary and physical activity behaviour during and after transition from working life to retirement. Results are inconsistent, with varying patterns of change being identified during the retirement transition dependent on study methodology. Most studies used single item questions and did not focus on different domains simultaneously (e.g. at work, during leisure time).[12 13] With regard to sedentary behaviour, both decreases in total sitting time and an increase in sedentary leisure activities such as watching TV have been reported. [12] With regard to physical activity, decreases were mainly seen in occupational physical activity and transport physical activity.[14] The domain in which the sedentary and physical activity behaviour is performed is important since determinants and health effects may be different.[15 16] To be able to design effective interventions, it is essential to know which sedentary and physical behaviour domains have potential to facilitate healthy aging during the retirement transition.

A previous study showed that changing from an active to a more sedentary occupation was compensated by exercising more during leisure time, and the other way around.[17] It is therefore conceivable that persons who retire from a physically active occupation might compensate by being more active during leisure time activities after retirement. To get deeper insight into behaviours related to physical activity after retirement, research that simultaneously investigates sedentary and physical activity behaviour in different domains both before and after retirement is warranted.

Our aim was to determine which sedentary and physical activity behaviours are predictors of unfavourable sedentary and physical activity behaviour after retirement. We analysed whether sedentary and physical activity behaviour after retirement can be predicted by pre-retirement

For peer review only - http://bmjopen.bmj.com/site/about/guidelines.xhtml

sedentary behaviour and physical activity in different domains (at work, at home or during leisure time) in a large population-based cohort including persons who recently retired. We hypothesized that having a more sedentary occupation, unfavourable leisure physical activity or unfavourable leisure sedentary behaviour before retirement predicts higher levels of sedentary behaviour and lower levels of physical activity behaviour after retirement. This study will add with valuable knowledge for public health and policy makers on who to target and which physical activity and sedentary behaviour to target to improve healthy aging during the retirement transition.

Methods

Study population

We used data from the Stockholm Public Health Cohort (SPHC), a large population-based cohort in Stockholm County.[18] Population samples were randomly selected from Statistics Sweden's Register of the total population, after stratification according to residential municipality. Every four years, participants from all three samples completed similar questionnaire-based surveys on a range of demographic- and health variables. Register data from Statistics Sweden have been linked to the self-reported information. The present study was approved by the Stockholm Regional Ethical Review Board (case number: 2016/749-32).

Of the participants who completed the survey in 2010, 67% completed the follow up survey in 2014.[18] Participants who reported information on physical activity and sedentary behaviour in both 2010 (baseline) and 2014 (follow-up) were assessed for eligibility (N=49133). Mean age of the sample assessed for eligibility was 54 years (SD=16), 57% were women and 48% highly educated. Participants were excluded if they were confined to bed on both occasions (n=26). In the current study, those who were employed or self-employed in 2010 but retired in 2014 were included, resulting in an analytical sample of 3272 participants.

Physical activity and sedentary behaviour assessment

Physical activity and sedentary behaviour were assessed with the physical activity questionnaire (PAQ), which has shown to be valid for classification into physically active or sedentary.[19 20] For all questions, participants were requested to answer on their average behaviour during the past 12 months, considering the week variability and seasonality.

Physical activity and sedentary in the following domains were assessed with this questionnaire:

- *Job activity*

For peer review only - http://bmjopen.bmj.com/site/about/guidelines.xhtml

With 6 answer categories: “mainly sedentary”, “sitting approximately half of the time”, “mainly standing”, “walking mostly, lifting, carrying a little”, “walking mainly, lifting, carrying a lot” or “heavy physical work”.

- *Sedentary leisure time*

This includes leisure sitting time e.g. watching TV or reading. There were 7 response categories ranging from less than one hour per day to more than 6 hours per day.

- *Physical activity at home*

This included physical activity during home, household and gardening tasks. There were 6 response categories ranging from less than 1 hr per day to more than 5 hrs per day.

- *Walking- and cycling activity*

With 6 response categories for answering combined walking and cycling activity, ranging from hardly ever to more than 2 hrs per day.

- *Exercise*

A question was asked about hours of exercise per week, excluding daily walking and cycling. Participants chose one of seven options ranging from hardly ever to more than 5 hours per week.

For peer review only

Data analyses

The cohort median for all physical activity and sedentary behaviour variables was determined and used to dichotomize behaviours into more favourable vs unfavourable. The medians were determined separately at baseline and at follow-up surveys, since occupational activity only applied to baseline and therefore the distribution of physical activity and sedentary behaviour domains was naturally different at follow-up after retirement.[21] Unfavourable sedentary behaviour at work (only baseline) was defined as mainly sedentary or sitting approximately half of the time. Unfavourable sedentary behaviour during leisure time at baseline was defined as 2-3 hours per day or more, and at follow-up as 3-4 hours per day or more. Physical activity at home was defined as unfavourable when less than 1 hr per day at baseline and less than 1-2 hours per day at follow-up. For both baseline and follow-up walking-and cycling activity were defined as unfavourable when performed for less than 20 minutes per day, and exercise when accumulating less than 1 hour per week.

Binary logistic regression analyses were conducted using general linear models in order to derive odds ratios (OR) of unfavourable behaviour after retirement for: 1) sedentary time, 2) physical activity at home, 3) walking/cycling and 4) exercise. Predictors were unfavourable behaviours before retirement related to: a) job activity, b) sedentary leisure time, c) physical activity at home, d) walking/cycling, and e) exercise.

For peer review only - http://bmjopen.bmj.com/site/about/guidelines.xhtml

A similar additional logistic regression analysis was conducted for all behaviours considered together, i.e. estimating the amount of unfavourable behaviours after retirement based on the amount of unfavourable behaviours before retirement. To obtain a dichotomous variable, the median number of unfavourable behaviours after retirement was used, i.e. 3 or more unfavourable behaviours.

Education was used as an indicator of socioeconomic position, and all models were adjusted for education and gender

We checked for multicollinearity by assessing both correlations between the predictors and with VIF statistics and concluded that there were no indications of collinearity. All statistical analyses were performed in IBM SPSS Statistics version 24 (Armonk, NY: IBM Corp).

Results

Mean age of the analytical sample at baseline was 63 years (SD=2), 55% were women and 43% highly educated. The associations between pre-retirement and after-retirement behaviours are shown in table 1.

The results can be summarized as:

- Unfavourable sedentary and physical activity behaviour in a certain domain before retirement was the strongest predictor of the same behaviour in the same domain after retirement.
- Unfavourable job physical activity predicted unfavourable sedentary time after retirement, but not any other unfavourable physical activity behaviour.
- Unfavourable exercise behaviour before retirement predicted all unfavourable sedentary and physical activity behaviours after retirement.

The relation between multiple unfavourable behaviours before and after retirement is shown descriptively in Table 2. Among participants who presented with all unfavourable behaviours at baseline the adjusted OR of having three or more unfavourable behaviour after retirement (vs. 2 or less) was 36.7 (95% CI=16.8-80.5). The corresponding ORs for 4 unfavourable behaviours before retirement was 14.3 (95% CI=7.1-29.0); 6.6 (95% CI=3.3-13.2) for 3 unfavourable behaviours; 3.2 (95% CI=1.6-6.5) for 2 unfavourable behaviours and 1.7 (95% CI=0.8-3.6) for 1 unfavourable behaviour.

Discussion

This longitudinal study increases the understanding of sedentary and physical activity behaviour during the retirement transition. We found that unfavourable pre-retirement physical activity and sedentary

For peer review only - http://bmjopen.bmj.com/site/about/guidelines.xhtml

For peer review only

behaviour at home or during leisure time were the strongest predictors of the same behaviour after retirement. Pre-retirement job activity did not predict low levels of physical activity but did predict sedentary behaviour after retirement. Furthermore, less than 1 hour exercise per week before retirement predicted all of the unfavourable behaviours after retirement. Moreover, the higher the number of unfavourable behaviours before retirement, the more likely it was that a person had multiple (at least three) unfavourable behaviours after retirement.

Contrary to our hypothesis, pre-retirement job activity did not seem to be related to post-retirement physical activity in this study, i.e. there was no hint that persons retiring from physically active jobs compensated the loss of this activity by increasing leisure time physical activity. A previous study showed that people changing job do compensate loss of job activity in their leisure time. [17] Retirement means a change to no job activity at all, potentially explaining why this compensation does not apply. Nevertheless, job activity did seem to predict sedentary behaviour after retirement in the anticipated direction. These results are in line with a previous study where decreases in occupational sedentary behaviour were compensated by increasing sitting time outside working hours.[22]

A systematic review[12] suggested that pre-retirement physical activity and sedentary behaviour outside work may be stronger predictors of behaviour after retirement compared to job activity. This hypothesis is supported by our findings that unfavourable pre-retirement physical activity and sedentary behaviour at home or during leisure time were the strongest predictors of the same unfavourable behaviours after retirement. These results confirm that behaviours tend to be rather stable over the life course [23], and this is probably true even after a major life change such as retirement. Furthermore, we found that unfavourable exercise behaviour before retirement seems to influence practically all unfavourable physical activity and sedentary behaviour after retirement. It is possible that regular exercise results in a higher physical fitness level, subsequently in a lower strain when performing physical activities, making it easier to maintain a favourable level even at older age. Therefore, interventions promoting exercise behaviour before retirement may potentially prevent unfavourable physical activity and sedentary behaviour after retirement.

Older adults often have unfavourable physical activity and sedentary behaviour [9 10] which negatively impacts healthy aging.[3-8] Our results imply that adults with multiple unfavourable pre-retirement behaviours are at higher risk to hold this profile after retirement. This group should be thought of as a priority for preventive interventions targeting physical activity. Despite the paucity of studies of interventions during the retirement transition, a review concluded that different types of counselling programs, such as group sessions; individual training sessions; in-home exercise programmes or e-health programs can lead to positive effects in aging adults.[24] Our results carry a decisive suggestion

For peer review only - http://bmjopen.bmj.com/site/about/guidelines.xhtml

that future studies should evaluate the effectiveness of pre-retirement exercise interventions on overall post-retirement physical activity and sedentary behaviour.

When interpreting the results of the study it should be realized that we used the sample medians to distinguish between favourable and unfavourable behaviour. Since there is no evidence for cut-off points for domain-specific physical activity and sedentary behaviour, it is unclear whether the medians reflect a true border between favourable and unfavourable. For job activity, we defined favourable as being more active at work. However, a recent review indicates that high levels of job activity might have detrimental health consequences and therefore not necessarily be favourable.[25] Furthermore, we did not separate different leisure time sedentary activities (such as computer use, TV, reading etc.) whilst certain leisure sitting time might be more likely to be combined with other unfavourable health behaviour such as drinking alcohol and eating snacks.[26]

Strengths and Limitations

To our knowledge, this is the largest cohort study that studied a variety of behaviours related to both physical activity and sedentary time in different domains both before and after retirement. However, there were also some limitations. First, all participants who retired in a time frame of 4 years were included in this study disregarding time since retirement, while this could have influenced behavioural adjustments. Second, we had no information on possible confounders of outcomes, such as comorbidities. Third, we included persons that completed two surveys, which might have led to a selection bias. A relatively large proportion of the included sample was highly educated, which is known to be related to more favourable exercise patterns and sedentary behaviour.[27-29] Furthermore, a decrease in income after retirement might have potentially influenced retirement behaviours including physical activity and sedentary behaviour. Last, we cannot rule out misclassification of sedentary behaviour and physical activity since it is known that self-report measures often demonstrate restricted validity and reliability.[30]

Conclusions

This study contributes to a deeper understanding of unhealthy aging with novel insights on unfavourable sedentary and physical activity behaviour after retirement. Despite the major life event of retirement, pre-retirement unfavourable behaviours seem likely to be carried into retirement age. Likewise, those with multiple unfavourable pre-retirement behaviours seem at a higher risk to hold the same unfavourable profile after retirement and should therefore be a priority target for preventive interventions. There was however no indication that persons retiring from physically active jobs compensated the loss of this activity by increasing leisure time physical activity. As unfavourable exercise behaviour before retirement predicted unfavourable sedentary and physical activity after

For peer review only - http://bmjopen.bmj.com/site/about/guidelines.xhtml
For peer review only

retirement in all domains, pre-retirement exercise interventions may have great potential to facilitate healthy aging.

For peer review only

For peer review only - http://bmjopen.bmj.com/site/about/guidelines.xhtml

Table 1. Baseline characteristics and predictors of more unfavourable sedentary and physical activity behaviour after retirement

	Sedentary leisure time			
	Unfavourable after retirement	Favourable after retirement		
	n=1441	n=1805		
*Predictors of unfavourable leisure time sedentary behaviour after retirement**	% (n)	% (n)	OR	95% CI
Unfavourable job activity before retirement	70 (988)	59 (1042)	**1.66**	**1.41-1.96**
Unfavourable sedentary leisure time before retirement	66 (933)	33 (588)	**4.19**	**3.59-4.89**
Unfavourable activity at home before retirement	36 (512)	29 (522)	**1.32**	**1.12-1.56**
Unfavourable walking/cycling before retirement	72 (1029)	67 (1194)	1.17	0.99-1.38
Unfavourable exercise before retirement	48 (678)	40 (709)	**1.29**	**1.10-1.50**

*adjusted for gender and education

	Physical activity at home			
	Unfavourable after retirement	Favourable after retirement		
	n=1928	n=1302		
*Predictors of unfavourable home physical activity after retirement**	% (n)	% (n)	OR	95% CI
Unfavourable job activity before retirement	66 (1252)	60 (769)	1.16	0.99-1.37
Unfavourable sedentary leisure time before retirement	50 (957)	44 (562)	**1.35**	**1.16-1.58**
Unfavourable activity at home before retirement	44 (837)	15 (188)	**3.92**	**3.26-4.72**
Unfavourable walking/cycling before retirement	71 (1359)	66 (850)	1.06	0.90-1.25
Unfavourable exercise before retirement	46 (878)	39 (503)	**1.23**	**1.05-1.44**

*adjusted for gender and education

For peer review only - http://bmjopen.bmj.com/site/about/guidelines.xhtml

For peer review only

	Walking/cycling			
	Unfavourable after retirement	Favourable after retirement		
	n=1923	n=1313		
*Predictors of unfavourable walking/cycling after retirement**	% (n)	% (n)	OR	95% CI
Unfavourable job activity before retirement	66 (1240)	61 (787)	1.07	0.91-1.26
Unfavourable sedentary leisure time before retirement	47 (902)	48 (620)	0.99	0.84-1.15
Unfavourable activity at home before retirement	34 (651)	29 (374)	1.04	0.88-1.23
Unfavourable walking/cycling before retirement	81 (1537)	53 (681)	**3.74**	**3.18-4.41**
Unfavourable exercise before retirement	48 (911)	37 (474)	**1.43**	**1.22-1.66**

*adjusted for gender and education

	Exercise			
	Unfavourable after retirement	Favourable after retirement		
	n=1414	n=1833		
*Predictors of unfavourable exercise after retirement**	% (n)	% (n)	OR	95% CI
Unfavourable job activity before retirement	65 (896)	63 (1134)	1.07	0.91-1.26
Unfavourable sedentary leisure time before retirement	52 (731)	44 (791)	**1.36**	**1.17-1.59**
Unfavourable activity at home before retirement	35 (483)	30 (547)	1.05	0.89-1.24
Unfavourable walking/cycling before retirement	73 (1021)	67 (1205)	1.15	0.97-1.36
Unfavourable exercise before retirement	64 (900)	26 (478)	**4.92**	**4.21-5.75**

*adjusted for gender and education

For peer review only - http://bmjopen.bmj.com/site/about/guidelines.xhtml

Table 2. Multiple unfavourable behaviours before and after retirement

		Number of unfavourable behaviours after retirement (n=3092)				
	% (n)	All	3	2	1	None
		10 (309)	24 (755)	35 (1087)	24 (732)	7 (209)
Number of unfavourable behaviours before retirement	All	43 (68)	35 (55)	18 (28)	4 (6)	0 (0)
	4	22 (109)	36 (182)	32 (160)	10 (50)	1 (4)
	3	9 (83)	30 (282)	39 (369)	19 (183)	3 (32)
	2	4 (40)	19 (170)	37 (340)	31 (286)	8 (74)
	1	2 (9)	12 (56)	35 (163)	35 (163)	16 (74)
	None	0 (0)	9 (10)	26 (27)	42 (44)	24 (25)

For peer review only

For peer review only - http://bmjopen.bmj.com/site/about/guidelines.xhtml

References

1. Organization WH. Global Health and Aging. Geneva: World Health Organization, 2011.
2. Dogra S, Stathokostas L. Sedentary behavior and physical activity are independent predictors of successful aging in middle-aged and older adults. *J Aging Res* 2012;2012:190654. doi: 10.1155/2012/190654 [published Online First: 2012/09/22]
3. Chau JY, Grunseit AC, Chey T, et al. Daily sitting time and all-cause mortality: a meta-analysis. *Plos One* 2013;8(11):e80000. doi: 10.1371/journal.pone.0080000

PONE-D-13-19234 [pii] [published Online First: 2013/11/16]
4. Wilmot EG, Edwardson CL, Achana FA, et al. Sedentary time in adults and the association with diabetes, cardiovascular disease and death: systematic review and meta-analysis. *Diabetologia* 2012;55(11):2895-905. doi: 10.1007/s00125-012-2677-z
5. Biswas A, Oh PI, Faulkner GE, et al. Sedentary time and its association with risk for disease incidence, mortality, and hospitalization in adults: a systematic review and meta-analysis. *Annals of internal medicine* 2015;162(2):123-32. doi: 10.7326/M14-1651
6. Bayan-Bravo A, Perez-Tasigchana RF, Lopez-Garcia E, et al. The association of major patterns of physical activity, sedentary behavior and sleeping with mortality in older adults. *J Sports Sci* 2018:1-10. doi: 10.1080/02640414.2018.1504617 [published Online First: 2018/08/02]
7. de Rezende LF, Rey-Lopez JP, Matsudo VK, et al. Sedentary behavior and health outcomes among older adults: a systematic review. *BMC Public Health* 2014;14:333. doi: 1471-2458-14-333 [pii]

10.1186/1471-2458-14-333 [published Online First: 2014/04/10]
8. Gennuso KP, Gangnon RE, Matthews CE, et al. Sedentary behavior, physical activity, and markers of health in older adults. *Med Sci Sports Exerc* 2013;45(8):1493-500. doi: 10.1249/MSS.0b013e318288a1e5 [published Online First: 2013/03/12]
9. Harvey JA, Chastin SF, Skelton DA. How Sedentary are Older People? A Systematic Review of the Amount of Sedentary Behavior. *J Aging Phys Act* 2015;23(3):471-87. doi: 2014-0164 [pii]

10.1123/japa.2014-0164 [published Online First: 2014/11/12]
10. Godfrey A, Lord S, Galna B, et al. The association between retirement and age on physical activity in older adults. *Age Ageing* 2014;43(3):386-93. doi: aft168 [pii]

10.1093/ageing/aft168 [published Online First: 2013/11/01]
11. Reichert FF, Barros AJ, Domingues MR, et al. The role of perceived personal barriers to engagement in leisure-time physical activity. *Am J Public Health* 2007;97(3):515-9. doi: 10.2105/AJPH.2005.070144 [published Online First: 2007/02/03]
12. Sprod J, Ferrar K, Olds T, et al. Changes in sedentary behaviours across the retirement transition: a systematic review. *Age Ageing* 2015;44(6):918-25. doi: afv140 [pii]

10.1093/ageing/afv140 [published Online First: 2015/10/28]
13. Barnett I, van Sluijs EM, Ogilvie D. Physical activity and transitioning to retirement: a systematic review. *Am J Prev Med* 2012;43(3):329-36. doi: S0749-3797(12)00394-7 [pii] [published Online First: 2012/08/18]
14. Barnett I, van Sluijs E, Ogilvie D, et al. Changes in household, transport and recreational physical activity and television viewing time across the transition to retirement: longitudinal evidence from the EPIC-Norfolk cohort. *J Epidemiol Community Health* 2014;68(8):747-53. doi: jech-2013-203225 [pii]

10.1136/jech-2013-203225 [published Online First: 2013/12/05]
15. Rhodes RE, Mark RS, Temmel CP. Adult sedentary behavior: a systematic review. *Am J Prev Med* 2012;42(3):e3-28. doi: 10.1016/j.amepre.2011.10.020

For peer review only - http://bmjopen.bmj.com/site/about/guidelines.xhtml

16. Ekelund U, Steene-Johannessen J, Brown WJ, et al. Does physical activity attenuate, or even eliminate, the detrimental association of sitting time with mortality? A harmonised meta-analysis of data from more than 1 million men and women. *Lancet* 2016;388(10051):1302-10. doi: 10.1016/S0140-6736(16)30370-1
17. Nooijen CFJ, Del Pozo-Cruz B, Nyberg G, et al. Are changes in occupational physical activity level compensated by changes in exercise behavior? *Eur J Public Health* 2018 doi: 10.1093/eurpub/cky007
18. Svensson AC, Fredlund P, Laflamme L, et al. Cohort profile: The Stockholm Public Health Cohort. *International journal of epidemiology* 2013;42(5):1263-72. doi: 10.1093/ije/dys126
19. Orsini N, Bellocco R, Bottai M, et al. Validity of self-reported total physical activity questionnaire among older women. *European journal of epidemiology* 2008;23(10):661-7. doi: 10.1007/s10654-008-9273-z
20. Norman A, Bellocco R, Bergstrom A, et al. Validity and reproducibility of self-reported total physical activity--differences by relative weight. *International journal of obesity and related metabolic disorders : journal of the International Association for the Study of Obesity* 2001;25(5):682-8. doi: 10.1038/sj.ijo.0801597
21. Olds T, Burton NW, Sprod J, et al. One day you'll wake up and won't have to go to work: The impact of changes in time use on mental health following retirement. *PLoS One* 2018;13(6):e0199605. doi: 10.1371/journal.pone.0199605
22. Mansoubi M, Pearson N, Biddle SJ, et al. Using Sit-to-Stand Workstations in Offices: Is There a Compensation Effect? *Med Sci Sports Exerc* 2016;48(4):720-5. doi: 10.1249/MSS.0000000000000802
23. Nooijen CFJ, Kallings LV, Blom V, et al. Common Perceived Barriers and Facilitators for Reducing Sedentary Behaviour among Office Workers. *Int J Environ Res Public Health* 2018;15(4) doi: 10.3390/ijerph15040792
24. Baxter S, Johnson M, Payne N, et al. Promoting and maintaining physical activity in the transition to retirement: a systematic review of interventions for adults around retirement age. *Int J Behav Nutr Phys Act* 2016;13:12. doi: 10.1186/s12966-016-0336-3
25. Coenen P, Huysmans MA, Holtermann A, et al. Do highly physically active workers die early? A systematic review with meta-analysis of data from 193 696 participants. *Br J Sports Med* 2018;52(20):1320-26. doi: 10.1136/bjsports-2017-098540
26. Nooijen CFJ, Moller J, Forsell Y, et al. Do unfavourable alcohol, smoking, nutrition and physical activity predict sustained leisure time sedentary behaviour? A population-based cohort study. *Prev Med* 2017;101:23-27. doi: S0091-7435(17)30182-2 [pii]

10.1016/j.ypmed.2017.05.019 [published Online First: 2017/05/23]
27. Chung S, Domino ME, Stearns SC, et al. Retirement and physical activity: analyses by occupation and wealth. *Am J Prev Med* 2009;36(5):422-8. doi: S0749-3797(09)00093-2 [pii]

10.1016/j.amepre.2009.01.026 [published Online First: 2009/03/10]
28. Shaw BA, Spokane LS. Examining the association between education level and physical activity changes during early old age. *J Aging Health* 2008;20(7):767-87. doi: 0898264308321081 [pii]

10.1177/0898264308321081 [published Online First: 2008/06/19]
29. Chad KE, Reeder BA, Harrison EL, et al. Profile of physical activity levels in community-dwelling older adults. *Med Sci Sports Exerc* 2005;37(10):1774-84. doi: 00005768-200510000-00019 [pii] [published Online First: 2005/11/02]
30. Prince SA, Adamo KB, Hamel ME, et al. A comparison of direct versus self-report measures for assessing physical activity in adults: a systematic review. *Int J Behav Nutr Phys Act* 2008;5:56. doi: 10.1186/1479-5868-5-56 [published Online First: 2008/11/08]

For peer review only - http://bmjopen.bmj.com/site/about/guidelines.xhtml

BMJ Open

Unfavourable sedentary and physical activity behaviour before and after retirement. A population-based cohort study

Journal:	*BMJ Open*
Manuscript ID	bmjopen-2020-037659.R1
Article Type:	Original research
Date Submitted by the Author:	03-May-2020
Complete List of Authors:	ter Hoeve, Nienke; Erasmus Univ, Rehabilitation Medicine<br>Ekblom, Maria; Swedish School of Sport and Health Sciences<br>Galanti, Maria; Karolinska Institutet<br>Forsell , Yvonne; Karolinska Institutet,<br>Nooijen, Carla; Swedish School of Sport and Health Sciences,
**Primary Subject Heading**:	Public health
Secondary Subject Heading:	Epidemiology
Keywords:	PUBLIC HEALTH, EPIDEMIOLOGY, PREVENTIVE MEDICINE

SCHOLARONE™
Manuscripts

I, the Submitting Author has the right to grant and does grant on behalf of all authors of the Work (as defined in the below author licence), an exclusive licence and/or a non-exclusive licence for contributions from authors who are: i) UK Crown employees; ii) where BMJ has agreed a CC-BY licence shall apply, and/or iii) in accordance with the terms applicable for US Federal Government officers or employees acting as part of their official duties; on a worldwide, perpetual, irrevocable, royalty-free basis to BMJ Publishing Group Ltd ("BMJ") its licensees and where the relevant Journal is co-owned by BMJ to the co-owners of the Journal, to publish the Work in this journal and any other BMJ products and to exploit all rights, as set out in our licence.

The Submitting Author accepts and understands that any supply made under these terms is made by BMJ to the Submitting Author unless you are acting as an employee on behalf of your employer or a postgraduate student of an affiliated institution which is paying any applicable article publishing charge ("APC") for Open Access articles. Where the Submitting Author wishes to make the Work available on an Open Access basis (and intends to pay the relevant APC), the terms of reuse of such Open Access shall be governed by a Creative Commons licence – details of these licences and which Creative Commons licence will apply to this Work are set out in our licence referred to above.

Other than as permitted in any relevant BMJ Author's Self Archiving Policies, I confirm this Work has not been accepted for publication elsewhere, is not being considered for publication elsewhere and does not duplicate material already published. I confirm all authors consent to publication of this Work and authorise the granting of this licence.

For peer review only

Unfavourable sedentary and physical activity behaviour before and after retirement. A population-based cohort study

Nienke ter Hoeve, Maria M. Ekblom, Rosaria Galanti, Yvonne Forsell, Carla F.J. Nooijen

Dr. Nienke ter Hoeve
Capri Cardiac Rehabilitation, Rotterdam, the Netherlands
The Department of Rehabilitation Medicine, Erasmus University Medical Center, Rotterdam, the Netherlands
n.terhoeve@erasmusmc.nl

Dr. Maria M. Ekblom
The Swedish School of Sport and Health Sciences (GIH), Stockholm, Sweden
The Department of Neuroscience, Karolinska Institutet, Stockholm, Sweden
maria.ekblom@gih.se

Prof. Rosaria Galanti
The Department of Global Public Health, Karolinska Institutet, Stockholm, Sweden
Centre for Epidemiology and Community Medicine, Stockholm County Council, Sweden
Rosaria.galanti@ki.se

Prof. Yvonne Forsell
The Department of Global Public Health, Karolinska Institutet, Stockholm, Sweden
Centre for Epidemiology and Community Medicine, Stockholm County Council, Sweden
Yvonne.Forsell@ki.se

Dr. Carla F.J. Nooijen (Corresponding author)
The Swedish School of Sport and Health Sciences (GIH), Stockholm, Sweden
The Department of Global Public Health, Karolinska Institutet, Stockholm, Sweden
carla.nooijen@gih.se

Corresponding author:
Carla Nooijen
Swedish School of Sport and Health Sciences (GIH)
Lidingövägen 1
Box 5626
SE-114 86 Stockholm
carla.nooijen@gih.se

Acknowledgements
Carla Nooijen is supported by a grant from The Swedish Research Council for Health, Working Life and Welfare: FORTE (2017-01385).

For peer review only

Abstract

Background: During transition to retirement there is often a re-arrangement of daily life which might provide a key opportunity for interventions to promote a non-sedentary and active lifestyle. To be able to design effective interventions, it is essential to know which sedentary and physical behaviour domains (e.g. at home or during leisure time) have potential to facilitate healthy aging during the retirement transition.

Objective: To determine whether unfavourable sedentary and physical activity behaviour before retirement predict unfavourable sedentary and physical activity behaviour after retirement.

Design: Population-based cohort

Setting and participants: Adults (n=3272) employed in 2010 but retired in 2014.

Methods: Self-reported pre-retirement job activity, sedentary leisure time, physical activity at home, walking-cycling and exercise, were assessed as predictors for unfavourable sedentary and physical activity behaviours after retirement using logistic regression. Unfavourable behaviours were defined based on the respective median of the cohort distribution. Furthermore, the odds ratio (OR) for having multiple unfavourable behaviours post-retirement was determined, based on the amount of unfavourable behaviours pre-retirement. All models were adjusted for gender and education.

Results: Unfavourable pre-retirement physical activity and sedentary behaviour at home or during leisure time were the strongest predictors of the same behaviours after retirement. Unfavourable job activity did not predict physical activity but did predict unfavourable sedentary behaviour after retirement (OR=1.66, 95% Confidence interval (CI)=1.41-1.96). Unfavourable exercise behaviour pre-retirement predicted unfavourable sedentary and physical activity post-retirement in all domains. With all behaviours being unfavourable pre-retirement, the OR of having at least 3 unfavourable behaviours post-retirement was 36.7 (95% CI=16.8-80.5).

Conclusions: Adults with a higher number of unfavourable pre-retirement physical activity and sedentary behaviours are likely to carry these unfavourable behaviours into retirement age. Interventions should target those with more unfavourable pre-retirement physical activity and sedentary behaviours pre-retirement, and those interventions focusing on exercise might have greatest potential

For peer review only - http://bmjopen.bmj.com/site/about/guidelines.xhtml

Strengths and limitations of the study

- The study described a large longitudinal cohort investigating changes in sedentary behaviour and physical activity during retirement transition.
- The used instrument (PAQ questionnaire) takes into account both physical activity and sedentary behaviour in different domains (e.g. at work, during leisure time).
- This study provides valuable knowledge for public health researchers and policy makers, indicating that interventions preferably focussing on exercise should target individuals with unfavourable physical activity and sedentary behaviours pre-retirement
- We did not have information on the exact time since retirement.

Funding statement

The work of Carla Nooijen was supported by The Swedish Research Council for Health, Working Life and Welfare grant number [FORTE (2017-01385)].

Competing interests: None declared.

Author's contribution

NtH and CN planned the study. NtH, ME, RG, YF, CN contributed to design and methodology of the manuscript. CN was responsible for data analysis and data interpretation. NtH, ME, RG, YF contributed to interpreting the data. NtH was responsible for drafting the manuscript and revision of the manuscript after review. All authors read and approved the final manuscript.

Data sharing

Applications for access to data can be send to the Stockholm County Council https://www.folkhalsoguiden.se/halsa-stockholm/halsa-stockholm---for-forskare/

Patient and public involvement

Patients or the public were not involved in the design, or conduct, or reporting, or dissemination plans of our research

For peer review only

Introduction

An aging population and increasing life expectancy result in a growing number of adults spending a long time in retirement.[1] Sedentary behaviour and physical activity are two related and independent predictors of healthy aging.[2] More sedentary time and less physical activity are related to increased risk of diabetes, cardiovascular disease, and all-cause mortality.[3-7] Studies in older adults have additionally shown that unfavourable sedentary and physical activity patterns increase the risk of functional limitations in the performance of activities of daily life such as walking and performing house chores.[8]

Elderly spend 65-80% of their waking hours sedentary and only a minority meets physical activity recommendations.[9,10] During transition to retirement, daily life often undergoes a re-arrangement which might provide a key opportunity for interventions to stimulate a non-sedentary and active lifestyle. Lack of time is a frequently reported barrier to physical activity, which might not exist any longer after retirement.[11]

For peer review only

Results of previous studies investigating changes in sedentary and physical activity behaviour during transition from working life to retirement identified varying patterns of change dependent on study methodology. Most studies used single item questions and did not focus on different domains simultaneously (e.g. at work, during leisure time).[12,13] With regard to sedentary behaviour, both decreases in total sitting time and an increase in sedentary leisure activities such as watching TV have been reported. [12,14,15] With regard to physical activity, decreases were mainly seen in occupational physical activity and transport physical activity[14,16], while increases were reported in time spent in leisure time physical activity and walking[15,17-19] The domain in which the sedentary and physical activity behaviour is performed is important since determinants and health effects may be different.[20,21] To be able to design effective interventions, it is essential to know which sedentary and physical behaviour domains have potential to facilitate healthy aging during the retirement transition.

A previous study showed that changing from an active to a less active occupation was compensated by exercising more during leisure time, and the other way around.[22] It is therefore conceivable that persons who retire from a physically active occupation might compensate by being more active during leisure time activities after retirement. In line with this hypothesis, it has been suggested that persons with a high occupational sitting time and low levels of physical activity before retirement are at risk for adverse sedentary behaviour outcomes after retirement.[14] Furthermore, a large cohort study found a relation between increased walking time and decreasing sedentary leisure time after retirement.[19] On the contrary, in another cohort study, no relations were found between changes in time spent in different physical activity and sedentary behaviours during retirement.[15] To get deeper insight into

For peer review only - http://bmjopen.bmj.com/site/about/guidelines.xhtml

behaviours related to sedentary behaviour and physical activity after retirement, more large-scale research that simultaneously investigates the relation between sedentary and physical activity behaviour in different domains both before and after retirement is warranted.

Our aim was to determine which sedentary and physical activity behaviours are predictors of unfavourable sedentary and physical activity behaviour after retirement. We analysed whether sedentary and physical activity behaviour after retirement can be predicted by pre-retirement sedentary behaviour and physical activity in different domains (at work, at home or during leisure time) in a large population-based cohort including persons who recently retired. We hypothesized that having a more sedentary occupation, unfavourable leisure physical activity or unfavourable leisure sedentary behaviour before retirement predicts higher levels of sedentary behaviour and lower levels of physical activity behaviour after retirement. This study will add with valuable knowledge for public health and policy makers on who to target and which physical activity and sedentary behaviour to target to improve healthy aging during the retirement transition.

Methods

Study population

We used data from the Stockholm Public Health Cohort (SPHC), a large population-based cohort in Stockholm County.[23] Population samples were randomly selected from Statistics Sweden's Register of the total population, after stratification according to residential municipality. Every four years, participants from all three samples completed similar questionnaire-based surveys on a range of demographic- and health variables. Register data from Statistics Sweden have been linked to the self-reported information. The present study was approved by the Stockholm Regional Ethical Review Board (case number: 2016/749-32).

Of the participants who completed the survey in 2010, 67% completed the follow up survey in 2014.[23] Participants who reported information on physical activity and sedentary behaviour in both 2010 (baseline) and 2014 (follow-up) were assessed for eligibility (N=49133). Mean age of the sample assessed for eligibility was 54 years (SD=16), 57% were women and 48% highly educated. Participants were excluded if they were confined to bed on both occasions (n=26). In the current study, those who were employed or self-employed in 2010 but full-time retired in 2014 were included, resulting in an analytical sample of 3272 participants. See Figure 1.

Physical activity and sedentary behaviour assessment

For peer review only - http://bmjopen.bmj.com/site/about/guidelines.xhtml

Physical activity and sedentary behaviour were assessed with the physical activity questionnaire (PAQ), which has shown to be valid for classification into physically active or sedentary.[24 25] For all questions, participants were requested to answer on their average behaviour during the past 12 months, considering the week variability and seasonality.

Physical activity and sedentary in the following domains were assessed with this questionnaire:

- *Job activity*

With 6 answer categories: "mainly sedentary", "sitting approximately half of the time", "mainly standing", "walking mostly, lifting, carrying a little", "walking mainly, lifting, carrying a lot" or "heavy physical work".

- *Sedentary leisure time*

This includes leisure sitting time e.g. watching TV or reading. There were 7 response categories ranging from less than one hour per day to more than 6 hours per day.

- *Physical activity at home*

This included physical activity during home, household and gardening tasks. There were 6 response categories ranging from less than 1 hr per day to more than 5 hrs per day.

- *Walking- and cycling activity*

With 6 response categories for answering combined walking and cycling activity, ranging from hardly ever to more than 2 hrs per day.

- *Exercise*

A question was asked about hours of exercise per week, excluding daily walking and cycling. Participants chose one of seven options ranging from hardly ever to more than 5 hours per week.

<u>Data analyses</u>

The cohort median for all physical activity and sedentary behaviour variables was determined and used to dichotomize behaviours into more favourable vs unfavourable. The medians were determined separately at baseline and at follow-up surveys, since occupational activity only applied to baseline and therefore the distribution of physical activity and sedentary behaviour domains was naturally different at follow-up after retirement.[26] Unfavourable job activity at work (only baseline) was defined as mainly sedentary or sitting approximately half of the time. Unfavourable sedentary behaviour during leisure time at baseline was defined as 2-3 hours per day or more, and at follow-up as 3-4 hours per day or more. Physical activity at home was defined as unfavourable when less than 1 hr per day at baseline and less than 1-2 hours per day at follow-up. For both baseline and follow-up walking-and cycling activity were defined as unfavourable when performed for less than 20 minutes per day, and exercise when accumulating less than 1 hour per week.

For peer review only - http://bmjopen.bmj.com/site/about/guidelines.xhtml

Binary logistic regression analyses were conducted using general linear models in order to derive odds ratios (OR) of unfavourable behaviour after retirement for: 1) sedentary time, 2) physical activity at home, 3) walking/cycling and 4) exercise. Predictors were unfavourable behaviours before retirement related to: a) job activity, b) sedentary leisure time, c) physical activity at home, d) walking/cycling, and e) exercise.

A similar additional logistic regression analysis was conducted for all behaviours considered together, i.e. estimating the amount of unfavourable behaviours after retirement based on the amount of unfavourable behaviours before retirement. To obtain a dichotomous variable, the median number of unfavourable behaviours after retirement was used, i.e. 3 or more unfavourable behaviours.

Education was used as an indicator of socioeconomic position, and all models were adjusted for education and gender

We checked for multicollinearity by assessing both correlations between the predictors and with VIF statistics and concluded that there were no indications of collinearity. All statistical analyses were performed in IBM SPSS Statistics version 24 (Armonk, NY: IBM Corp).

Results

Mean age of the analytical sample at baseline was 63 years (SD=2), 55% were women and 43% highly educated. The associations between pre-retirement and after-retirement behaviours are shown in Table 1.

The results can be summarized as:

- Unfavourable pre-retirement physical activity and sedentary behaviour at home or during leisure time were the strongest predictors of the same behaviour after retirement.
- Unfavourable job physical activity predicted unfavourable sedentary time after retirement, but not any other unfavourable physical activity behaviour.
- Unfavourable exercise behaviour before retirement predicted all unfavourable sedentary and physical activity behaviours after retirement.

-Unfavourable sedentary leisure time predicted all unfavourable behaviours, except for walking/cycling

The relation between multiple unfavourable behaviours before and after retirement is shown descriptively in Table 2. Among participants who presented with all unfavourable behaviours at

For peer review only

baseline the adjusted OR of having three or more unfavourable behaviour after retirement (vs. 2 or less) was 36.7 (95% CI=16.8-80.5). The corresponding ORs for 4 unfavourable behaviours before retirement was 14.3 (95% CI=7.1-29.0); 6.6 (95% CI=3.3-13.2) for 3 unfavourable behaviours; 3.2 (95% CI=1.6-6.5) for 2 unfavourable behaviours and 1.7 (95% CI=0.8-3.6) for 1 unfavourable behaviour.

Discussion

This longitudinal study increases the understanding of sedentary and physical activity behaviour during the retirement transition. We found that unfavourable pre-retirement physical activity and sedentary behaviour at home or during leisure time were the strongest predictors of the same behaviour after retirement. Pre-retirement job activity did not predict low levels of physical activity but did predict sedentary behaviour after retirement. Furthermore, less than 1 hour exercise per week before retirement predicted all of the unfavourable behaviours after retirement. Unfavourable sedentary leisure time before retirement predicted both sedentary time and most physical activity behaviours after retirement**.** Moreover, the higher the number of unfavourable behaviours before retirement, the more likely it was that a person had multiple (at least three) unfavourable behaviours after retirement.

Contrary to our hypothesis, pre-retirement job activity did not seem to be related to post-retirement physical activity in this study, i.e. there was no hint that persons retiring from physically active jobs compensated the loss of this activity by increasing leisure time physical activity. A previous study showed that people changing job do compensate loss of job activity in their leisure time. [22] Retirement means a change to no job activity at all, potentially explaining why this compensation does not apply. Nevertheless, job activity did seem to predict sedentary behaviour after retirement in the anticipated direction. These results are in line with a previous study where decreases in occupational sedentary behaviour were compensated by increasing sitting time outside working hours.[27]

A systematic review[12] suggested that pre-retirement physical activity and sedentary behaviour outside work may be stronger predictors of behaviour after retirement compared to job activity. This hypothesis is supported by our findings that unfavourable pre-retirement physical activity and sedentary behaviour at home or during leisure time were the strongest predictors of the same unfavourable behaviours after retirement. These results confirm that behaviours tend to be rather stable over the life course [28], and this is probably true even after a major life change such as retirement.

We found that unfavourable exercise behaviour before retirement seems to influence practically all unfavourable physical activity and sedentary behaviours after retirement. It is possible that regular

For peer review only - http://bmjopen.bmj.com/site/about/guidelines.xhtml

exercise results in a higher physical fitness level, subsequently in a lower strain when performing physical activities, making it easier to maintain a favourable level of both physical activity and sedentary behaviour even at older age. This outcome is in line with a previous study that found that persons with low levels of physical activity before retirement are at risk for unfavourable sedentary behaviour after retirement.[14] In addition to exercise behaviour, unfavourable sedentary leisure time before retirement predicted both sedentary time and most physical activity behaviours after retirement**.** These outcomes suggest that interventions promoting exercise behaviour and limiting sedentary time before retirement may potentially prevent unfavourable physical activity and sedentary behaviour also after retirement.

Older adults often have unfavourable physical activity and sedentary behaviour [9 10] which negatively impacts healthy aging.[3-8] Our results imply that adults with multiple unfavourable pre-retirement behaviours are at higher risk to hold this profile after retirement. This group should be thought of as a priority for preventive interventions targeting physical activity and sedentary behaviour. Despite the paucity of studies of interventions during the retirement transition, a review concluded that different types of counselling programs, such as group sessions; individual training sessions; in-home exercise programmes or e-health programs can lead to positive effects in aging adults.[29] Our results carry a decisive suggestion that future studies should evaluate the effectiveness of pre-retirement exercise and sedentary behaviour interventions on overall post-retirement physical activity and sedentary behaviour.

When interpreting the results of the study it should be realized that we used the sample medians to distinguish between favourable and unfavourable behaviour. Since there is no evidence for cut-off points for domain-specific physical activity and sedentary behaviour, it is unclear whether the medians reflect a true border between favourable and unfavourable. The question on job activity was a combination of sedentary behaviour and physical activity and our definition of favourable was being more active at work. However, a recent review indicates that high levels of job activity might have detrimental health consequences and therefore not necessarily be favourable.[30] Furthermore, we did not separate different leisure time sedentary activities (such as computer use, TV, reading etc.) whilst certain leisure sitting time might be more likely to be combined with other unfavourable health behaviours such as drinking alcohol and eating snacks.[31] In addition, we did also not separate between walking and cycling and between different exercise modalities even though we are aware that health effects might depend on the intensity and type of exercise performed.

Strengths and Limitations

For peer review only - http://bmjopen.bmj.com/site/about/guidelines.xhtml

The used instrument (PAQ questionnaire) takes into account both physical activity and sedentary behaviour in different domains and was used to measure these behaviours both before and after retirement, which is a major strength of our study. Nevertheless, as discussed above, the instrument does not differentiate between vocational physical activity and sedentary behaviour, between different types of leisure sedentary activities and between types of exercise performed. There were also some limitations. First, all participants who retired in a time frame of 4 years were included in this study. There was no information available on the exact time of retirement, while this could have influenced behavioural adjustments. Second, we had no information on possible confounders of outcomes, such as comorbidities. Third, we included persons that completed two surveys, which might have led to a selection bias. A relatively large proportion of the included sample was highly educated, which is known to be related to more favourable exercise patterns and sedentary behaviour.[32-34] Furthermore, a decrease in income after retirement might have potentially influenced retirement behaviours including physical activity and sedentary behaviour. Last, we cannot rule out misclassification of sedentary behaviour and physical activity since it is known that self-report measures often demonstrate restricted validity and reliability.[35]

Conclusions

This study contributes to a deeper understanding of unhealthy aging with novel insights on unfavourable sedentary and physical activity behaviour after retirement. Despite the major life event of retirement, pre-retirement unfavourable behaviours seem likely to be carried into retirement age. Likewise, those with multiple unfavourable pre-retirement behaviours seem at a higher risk to hold the same unfavourable profile after retirement. There was no indication that persons retiring from physically active jobs compensated the loss of this activity by increasing leisure time physical activity. Interventions should target those with more unfavourable pre-retirement physical activity and sedentary behaviours pre-retirement, and those interventions focusing on exercise might have greatest potential.

For peer review only - http://bmjopen.bmj.com/site/about/guidelines.xhtml

Table 1. Baseline characteristics and predictors of more unfavourable sedentary and physical activity behaviour after retirement

	**Sedentary leisure time after retirement**			
	Unfavourable after retirement	Favourable after retirement		
	n=1441	n=1805		
*Predictors of unfavourable leisure time sedentary behaviour after retirement**	% (n)	% (n)	OR	95% CI
Unfavourable job activity before retirement	70 (988)	59 (1042)	**1.66**	**1.41-1.96**
Unfavourable sedentary leisure time before retirement	66 (933)	33 (588)	**4.19**	**3.59-4.89**
Unfavourable activity at home before retirement	36 (512)	29 (522)	**1.32**	**1.12-1.56**
Unfavourable walking/cycling before retirement	72 (1029)	67 (1194)	1.17	0.99-1.38
Unfavourable exercise before retirement	48 (678)	40 (709)	**1.29**	**1.10-1.50**

*adjusted for gender and education

	**Physical activity at home after retirement**			
	Unfavourable after retirement	Favourable after retirement		
	n=1928	n=1302		
*Predictors of unfavourable home physical activity after retirement**	% (n)	% (n)	OR	95% CI
Unfavourable job activity before retirement	66 (1252)	60 (769)	1.16	0.99-1.37
Unfavourable sedentary leisure time before retirement	50 (957)	44 (562)	**1.35**	**1.16-1.58**
Unfavourable activity at home before retirement	44 (837)	15 (188)	**3.92**	**3.26-4.72**
Unfavourable walking/cycling before retirement	71 (1359)	66 (850)	1.06	0.90-1.25
Unfavourable exercise before retirement	46 (878)	39 (503)	**1.23**	**1.05-1.44**

*adjusted for gender and education

For peer review only

For peer review only - http://bmjopen.bmj.com/site/about/guidelines.xhtml

	Walking/cycling after retirement			
	Unfavourable after retirement	Favourable after retirement		
	n=1923	n=1313		
*Predictors of unfavourable walking/cycling after retirement**	% (n)	% (n)	OR	95% CI
Unfavourable job activity before retirement	66 (1240)	61 (787)	1.07	0.91-1.26
Unfavourable sedentary leisure time before retirement	47 (902)	48 (620)	0.99	0.84-1.15
Unfavourable activity at home before retirement	34 (651)	29 (374)	1.04	0.88-1.23
Unfavourable walking/cycling before retirement	81 (1537)	53 (681)	**3.74**	**3.18-4.41**
Unfavourable exercise before retirement	48 (911)	37 (474)	**1.43**	**1.22-1.66**

*adjusted for gender and education

	Exercise after retirement			
	Unfavourable after retirement	Favourable after retirement		
	n=1414	n=1833		
*Predictors of unfavourable exercise after retirement**	% (n)	% (n)	OR	95% CI
Unfavourable job activity before retirement	65 (896)	63 (1134)	1.07	0.91-1.26
Unfavourable sedentary leisure time before retirement	52 (731)	44 (791)	**1.36**	**1.17-1.59**
Unfavourable activity at home before retirement	35 (483)	30 (547)	1.05	0.89-1.24
Unfavourable walking/cycling before retirement	73 (1021)	67 (1205)	1.15	0.97-1.36
Unfavourable exercise before retirement	64 (900)	26 (478)	**4.92**	**4.21-5.75**

*adjusted for gender and education

For peer review only

Table 2. Multiple unfavourable behaviours before and after retirement

		Number of unfavourable behaviours after retirement (n=3092)				
	% (n)	All	3	2	1	None
		10 (309)	24 (755)	35 (1087)	24 (732)	7 (209)
Number of unfavourable behaviours before retirement	All	43 (68)	35 (55)	18 (28)	4 (6)	0 (0)
	4	22 (109)	36 (182)	32 (160)	10 (50)	1 (4)
	3	9 (83)	30 (282)	39 (369)	19 (183)	3 (32)
	2	4 (40)	19 (170)	37 (340)	31 (286)	8 (74)
	1	2 (9)	12 (56)	35 (163)	35 (163)	16 (74)
	None	0 (0)	9 (10)	26 (27)	42 (44)	24 (25)

For peer review only

Figure 1. Flowchart of Participants

For peer review only

For peer review only - http://bmjopen.bmj.com/site/about/guidelines.xhtml

References

1. Organization WH. Global Health and Aging. Geneva: World Health Organization, 2011.
2. Dogra S, Stathokostas L. Sedentary behavior and physical activity are independent predictors of successful aging in middle-aged and older adults. *J Aging Res* 2012;2012:190654. doi: 10.1155/2012/190654 [published Online First: 2012/09/22]
3. Chau JY, Grunseit AC, Chey T, et al. Daily sitting time and all-cause mortality: a meta-analysis. *Plos One* 2013;8(11):e80000. doi: 10.1371/journal.pone.0080000

PONE-D-13-19234 [pii] [published Online First: 2013/11/16]
4. Wilmot EG, Edwardson CL, Achana FA, et al. Sedentary time in adults and the association with diabetes, cardiovascular disease and death: systematic review and meta-analysis. *Diabetologia* 2012;55(11):2895-905. doi: 10.1007/s00125-012-2677-z
5. Biswas A, Oh PI, Faulkner GE, et al. Sedentary time and its association with risk for disease incidence, mortality, and hospitalization in adults: a systematic review and meta-analysis. *Annals of internal medicine* 2015;162(2):123-32. doi: 10.7326/M14-1651
6. Bayan-Bravo A, Perez-Tasigchana RF, Lopez-Garcia E, et al. The association of major patterns of physical activity, sedentary behavior and sleeping with mortality in older adults. *J Sports Sci* 2018:1-10. doi: 10.1080/02640414.2018.1504617 [published Online First: 2018/08/02]
7. de Rezende LF, Rey-Lopez JP, Matsudo VK, et al. Sedentary behavior and health outcomes among older adults: a systematic review. *BMC Public Health* 2014;14:333. doi: 1471-2458-14-333 [pii]

10.1186/1471-2458-14-333 [published Online First: 2014/04/10]
8. Gennuso KP, Gangnon RE, Matthews CE, et al. Sedentary behavior, physical activity, and markers of health in older adults. *Med Sci Sports Exerc* 2013;45(8):1493-500. doi: 10.1249/MSS.0b013e318288a1e5 [published Online First: 2013/03/12]
9. Harvey JA, Chastin SF, Skelton DA. How Sedentary are Older People? A Systematic Review of the Amount of Sedentary Behavior. *J Aging Phys Act* 2015;23(3):471-87. doi: 2014-0164 [pii]

10.1123/japa.2014-0164 [published Online First: 2014/11/12]
10. Godfrey A, Lord S, Galna B, et al. The association between retirement and age on physical activity in older adults. *Age Ageing* 2014;43(3):386-93. doi: aft168 [pii]

10.1093/ageing/aft168 [published Online First: 2013/11/01]
11. Reichert FF, Barros AJ, Domingues MR, et al. The role of perceived personal barriers to engagement in leisure-time physical activity. *Am J Public Health* 2007;97(3):515-9. doi: 10.2105/AJPH.2005.070144 [published Online First: 2007/02/03]
12. Sprod J, Ferrar K, Olds T, et al. Changes in sedentary behaviours across the retirement transition: a systematic review. *Age Ageing* 2015;44(6):918-25. doi: afv140 [pii]

10.1093/ageing/afv140 [published Online First: 2015/10/28]
13. Barnett I, van Sluijs EM, Ogilvie D. Physical activity and transitioning to retirement: a systematic review. *Am J Prev Med* 2012;43(3):329-36. doi: S0749-3797(12)00394-7 [pii] [published Online First: 2012/08/18]
14. Leskinen T, Pulakka A, Heinonen OJ, et al. Changes in non-occupational sedentary behaviours across the retirement transition: the Finnish Retirement and Aging (FIREA) study. *J Epidemiol Community Health* 2018;72(8):695-701. doi: 10.1136/jech-2017-209958 [published Online First: 2018/04/11]
15. Menai M, Fezeu L, Charreire H, et al. Changes in sedentary behaviours and associations with physical activity through retirement: a 6-year longitudinal study. *PLoS One* 2014;9(9):e106850. doi: 10.1371/journal.pone.0106850 [published Online First: 2014/09/27]
16. Barnett I, van Sluijs E, Ogilvie D, et al. Changes in household, transport and recreational physical activity and television viewing time across the transition to retirement: longitudinal evidence

For peer review only - http://bmjopen.bmj.com/site/about/guidelines.xhtml

from the EPIC-Norfolk cohort. *J Epidemiol Community Health* 2014;68(8):747-53. doi: jech-2013-203225 [pii]

10.1136/jech-2013-203225 [published Online First: 2013/12/05]

17. Jones SA, Li Q, Aiello AE, et al. Physical Activity, Sedentary Behavior, and Retirement: The Multi-Ethnic Study of Atherosclerosis. *Am J Prev Med* 2018;54(6):786-94. doi: 10.1016/j.amepre.2018.02.022 [published Online First: 2018/04/14]
18. Holstila A, Manty M, Rahkonen O, et al. Statutory retirement and changes in self-reported leisure-time physical activity: a follow-up study with three time-points. *BMC Public Health* 2017;17(1):528. doi: 10.1186/s12889-017-4455-9 [published Online First: 2017/06/01]
19. Touvier M, Bertrais S, Charreire H, et al. Changes in leisure-time physical activity and sedentary behaviour at retirement: a prospective study in middle-aged French subjects. *Int J Behav Nutr Phys Act* 2010;7:14. doi: 10.1186/1479-5868-7-14 [published Online First: 2010/02/26]
20. Rhodes RE, Mark RS, Temmel CP. Adult sedentary behavior: a systematic review. *Am J Prev Med* 2012;42(3):e3-28. doi: 10.1016/j.amepre.2011.10.020
21. Ekelund U, Steene-Johannessen J, Brown WJ, et al. Does physical activity attenuate, or even eliminate, the detrimental association of sitting time with mortality? A harmonised meta-analysis of data from more than 1 million men and women. *Lancet* 2016;388(10051):1302-10. doi: S0140-6736(16)30370-1 [pii]

10.1016/S0140-6736(16)30370-1 [published Online First: 2016/08/01]

22. Nooijen CFJ, Del Pozo-Cruz B, Nyberg G, et al. Are changes in occupational physical activity level compensated by changes in exercise behavior? *Eur J Public Health* 2018 doi: 4827075 [pii]

10.1093/eurpub/cky007 [published Online First: 2018/02/01]

23. Svensson AC, Fredlund P, Laflamme L, et al. Cohort profile: The Stockholm Public Health Cohort. *International journal of epidemiology* 2013;42(5):1263-72. doi: 10.1093/ije/dys126
24. Orsini N, Bellocco R, Bottai M, et al. Validity of self-reported total physical activity questionnaire among older women. *European journal of epidemiology* 2008;23(10):661-7. doi: 10.1007/s10654-008-9273-z
25. Norman A, Bellocco R, Bergstrom A, et al. Validity and reproducibility of self-reported total physical activity--differences by relative weight. *International journal of obesity and related metabolic disorders : journal of the International Association for the Study of Obesity* 2001;25(5):682-8. doi: 10.1038/sj.ijo.0801597
26. Olds T, Burton NW, Sprod J, et al. One day you'll wake up and won't have to go to work: The impact of changes in time use on mental health following retirement. *PLoS One* 2018;13(6):e0199605. doi: 10.1371/journal.pone.0199605
27. Mansoubi M, Pearson N, Biddle SJ, et al. Using Sit-to-Stand Workstations in Offices: Is There a Compensation Effect? *Med Sci Sports Exerc* 2016;48(4):720-5. doi: 10.1249/MSS.0000000000000802
28. Nooijen CFJ, Kallings LV, Blom V, et al. Common Perceived Barriers and Facilitators for Reducing Sedentary Behaviour among Office Workers. *Int J Environ Res Public Health* 2018;15(4) doi: ijerph15040792 [pii] 10.3390/ijerph15040792 [published Online First: 2018/04/20]

29. Baxter S, Johnson M, Payne N, et al. Promoting and maintaining physical activity in the transition to retirement: a systematic review of interventions for adults around retirement age. *Int J Behav Nutr Phys Act* 2016;13:12. doi: 10.1186/s12966-016-0336-3
30. Coenen P, Huysmans MA, Holtermann A, et al. Do highly physically active workers die early? A systematic review with meta-analysis of data from 193 696 participants. *Br J Sports Med* 2018;52(20):1320-26. doi: 10.1136/bjsports-2017-098540
31. Nooijen CFJ, Moller J, Forsell Y, et al. Do unfavourable alcohol, smoking, nutrition and physical activity predict sustained leisure time sedentary behaviour? A population-based cohort study. *Prev Med* 2017;101:23-27. doi: S0091-7435(17)30182-2 [pii]

For peer review only

For peer review only - http://bmjopen.bmj.com/site/about/guidelines.xhtml

10.1016/j.ypmed.2017.05.019 [published Online First: 2017/05/23]

32. Chung S, Domino ME, Stearns SC, et al. Retirement and physical activity: analyses by occupation and wealth. *Am J Prev Med* 2009;36(5):422-8. doi: S0749-3797(09)00093-2 [pii]

10.1016/j.amepre.2009.01.026 [published Online First: 2009/03/10]

33. Shaw BA, Spokane LS. Examining the association between education level and physical activity changes during early old age. *J Aging Health* 2008;20(7):767-87. doi: 0898264308321081 [pii]

10.1177/0898264308321081 [published Online First: 2008/06/19]

34. Chad KE, Reeder BA, Harrison EL, et al. Profile of physical activity levels in community-dwelling older adults. *Med Sci Sports Exerc* 2005;37(10):1774-84. doi: 00005768-200510000-00019 [pii] [published Online First: 2005/11/02]

35. Prince SA, Adamo KB, Hamel ME, et al. A comparison of direct versus self-report measures for assessing physical activity in adults: a systematic review. *Int J Behav Nutr Phys Act* 2008;5:56. doi: 10.1186/1479-5868-5-56 [published Online First: 2008/11/08]

For peer review only

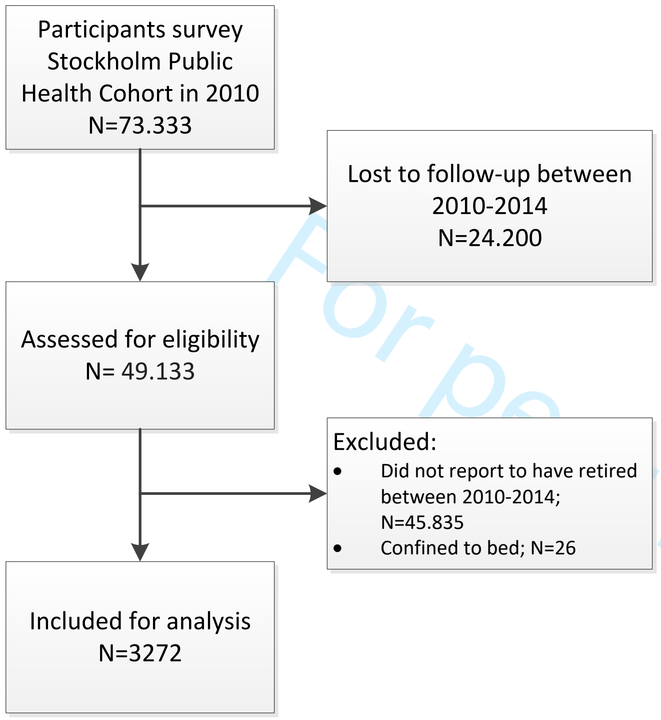

For peer review only

STROBE Statement—Checklist of items that should be included in reports of ***cohort studies***

	Item No	Recommendation
**Title and abstract**	1	(*a*) Indicate the study's design with a commonly used term in the title or the abstract **p.2 (marked copy)**
		(*b*) Provide in the abstract an informative and balanced summary of what was done and what was found **p.2 (marked copy)**
**Introduction**		
Background/rationale	2	Explain the scientific background and rationale for the investigation being reported **p.4 (marked copy)**
Objectives	3	State specific objectives, including any prespecified hypotheses **p.5 (marked copy)**
**Methods**		
Study design	4	Present key elements of study design early in the paper **p.5 (marked copy)**
Setting	5	Describe the setting, locations, and relevant dates, including periods of recruitment, exposure, follow-up, and data collection **p.5 (marked copy)**
Participants	6	(*a*) Give the eligibility criteria, and the sources and methods of selection of participants. Describe methods of follow-up. **p.5 (marked copy)**
		(*b*) For matched studies, give matching criteria and number of exposed and unexposed **N.A.**
Variables	7	Clearly define all outcomes, exposures, predictors, potential confounders, and effect modifiers. Give diagnostic criteria, if applicable **p.6-7 (marked copy)**
Data sources/ measurement	8*	For each variable of interest, give sources of data and details of methods of assessment (measurement). Describe comparability of assessment methods if there is more than one group **p.6 (marked copy)**
Bias	9	Describe any efforts to address potential sources of bias **p.7 + p.10 (marked copy)**
Study size	10	Explain how the study size was arrived at **p.5 (marked copy) + Figure 1**
Quantitative variables	11	Explain how quantitative variables were handled in the analyses. If applicable, describe which groupings were chosen and why **p.6-7 (marked copy)**
Statistical methods	12	(*a*) Describe all statistical methods, including those used to control for confounding **p.6-7 (marked copy)**
		(*b*) Describe any methods used to examine subgroups and interactions **p.6-7 (marked copy)**
		(*c*) Explain how missing data were addressed **N.A. complete cases selected, see page 5**
		(*d*) If applicable, explain how loss to follow-up was addressed **p.5 (marked copy)**
		(*e*) Describe any sensitivity analyses **N.a.**
**Results**		
Participants	13*	(a) Report numbers of individuals at each stage of study—eg numbers potentially eligible, examined for eligibility, confirmed eligible, included in the study, completing follow-up, and analysed **p.5 (marked copy) + Figure 1**
		(b) Give reasons for non-participation at each stage **N.a.**
		(c) Consider use of a flow diagram **Figure 1**
Descriptive data	14*	(a) Give characteristics of study participants (eg demographic, clinical, social) and information on exposures and potential confounders **p.7 (marked copy)**
		(b) Indicate number of participants with missing data for each variable of interest **N.A. complete cases selected, see page 5**
		(c) Summarise follow-up time (eg, average and total amount) **N.A.**
Outcome data	15*	Report numbers of outcome events or summary measures over time **p.7+8 (marked**

For peer review only

		**copy)**
Main results	16	(*a*) Give unadjusted estimates and, if applicable, confounder-adjusted estimates and their precision (eg, 95% confidence interval). Make clear which confounders were adjusted for and why they were included **p.8 + Table 1+2 (marked copy)**
		(*b*) Report category boundaries when continuous variables were categorized **p.6+7 (marked copy)**
		(*c*) If relevant, consider translating estimates of relative risk into absolute risk for a meaningful time period **N.a.**
Other analyses	17	Report other analyses done—eg analyses of subgroups and interactions, and sensitivity analyses **N.a.**
**Discussion**		
Key results	18	Summarise key results with reference to study objectives **p.8 (marked copy)**
Limitations	19	Discuss limitations of the study, taking into account sources of potential bias or imprecision. Discuss both direction and magnitude of any potential bias **p.10 (marked copy)**
Interpretation	20	Give a cautious overall interpretation of results considering objectives, limitations, multiplicity of analyses, results from similar studies, and other relevant evidence **p.10+11 (marked copy)**
Generalisability	21	Discuss the generalisability (external validity) of the study results **p.8-11 (marked copy)**
**Other information**		
Funding	22	Give the source of funding and the role of the funders for the present study and, if applicable, for the original study on which the present article is based **p.3 (marked copy)**

*Give information separately for exposed and unexposed groups.

Note: An Explanation and Elaboration article discusses each checklist item and gives methodological background and published examples of transparent reporting. The STROBE checklist is best used in conjunction with this article (freely available on the Web sites of PLoS Medicine at http://www.plosmedicine.org/, Annals of Internal Medicine at http://www.annals.org/, and Epidemiology at http://www.epidem.com/). Information on the STROBE Initiative is available at http://www.strobe-statement.org.

For peer review only
